# Pacinian corpuscle loss on routine forefoot MRI as an imaging biomarker for large-fiber sensory dysfunction in type 2 diabetic polyneuropathy

**DOI:** 10.1007/s00330-026-12576-7

**Published:** 2026-04-29

**Authors:** Sophia Samira Goller, Georg Wilhelm Kajdi, Christoph Germann, Martin Schubert, Michèle Hubli, Felix Wilhelm Arnaud Waibel, Reto Sutter

**Affiliations:** 1 Department of Radiology, Balgrist University Hospital, Faculty of Medicine, University of Zurich, Zurich, Switzerland; 2 Spinal Cord Injury Center, Balgrist University Hospital, Faculty of Medicine, University of Zurich, Zurich, Switzerland; 3 Department of Orthopaedics, Balgrist University Hospital, Faculty of Medicine, University of Zurich, Zurich, Switzerland

**Keywords:** Diabetes, Diabetic sensorimotor polyneuropathy, MRI, Nerve conduction studies, Pacinian corpuscle

## Abstract

**Objectives:**

To evaluate the relationship between Pacinian corpuscle (PC) count on forefoot MRI in diabetic sensorimotor polyneuropathy (DSP) and large-fiber sensory dysfunction as quantified by nerve conduction studies (NCS).

**Materials and methods:**

Thirty-nine patients with type 2 diabetes and neurologically confirmed DSP (mean age 67.9 ± 13.5 years; 29 males) underwent forefoot MRI and NCS, including compound muscle and sensory nerve action potentials (cMAP, sNAP) and conduction velocities of tibial, peroneal, and sural nerves. PC counts were assessed in the subcutaneous and deep regions of each digit. Spearman’s rank correlation analysis examined the relationship between total PC counts and sural sNAP amplitudes.

**Results:**

Based on motor and sensory amplitudes and age-adjusted conduction velocities of NCS, 10 patients were classified as having mild-to-moderate DSP, and 29 as having severe DSP. Severe DSP was associated with sensory large-fiber impairment, with 86.1% of patients showing abolished sural sNAP amplitudes. Total PC counts were significantly lower in severe cases compared to mild-to-moderate polyneuropathy (54.1 ± 40.6 vs 146.1 ± 43.2; *p* < 0.001). Spearman’s rank correlation analysis revealed a strong positive association between PC counts and sural sNAP amplitudes (ρ = 0.638, *p* < 0.001). Likewise, patients with severe DSP had substantially reduced or absent sural sNAP amplitudes (median [range]: 0.1 [0.1–1.7] µV) and lower PC counts, while those with mild-to-moderate DSP showed both higher sural sNAP amplitudes (median [range]: 2.3 [0.9–11.4] µV) and PC counts (*p* < 0.001).

**Conclusion:**

Forefoot MRI-detected PC counts closely correlate with large-fiber sensory function in DSP, supporting their potential as noninvasive imaging biomarkers of polyneuropathy severity.

**Key Points:**

***Question*** *DSP is linked to loss of PC on forefoot MRI; however, it remains unclear whether the number of PC correlates with DSP severity*.

***Findings*** *Forefoot MRI showed lower PC counts in patients with severe DSP than in those with mild-to-moderate disease and indicated a strong association between PC loss and electrophysiological measures of large-fiber sensory dysfunction*.

***Clinical relevance*** *MRI-based PC quantification is a promising imaging biomarker for large-fiber sensory dysfunction in DSP, complementing established clinical and electrophysiological methods for diagnosing and assessing DSP severity*.

**Graphical Abstract:**

**Figure:**
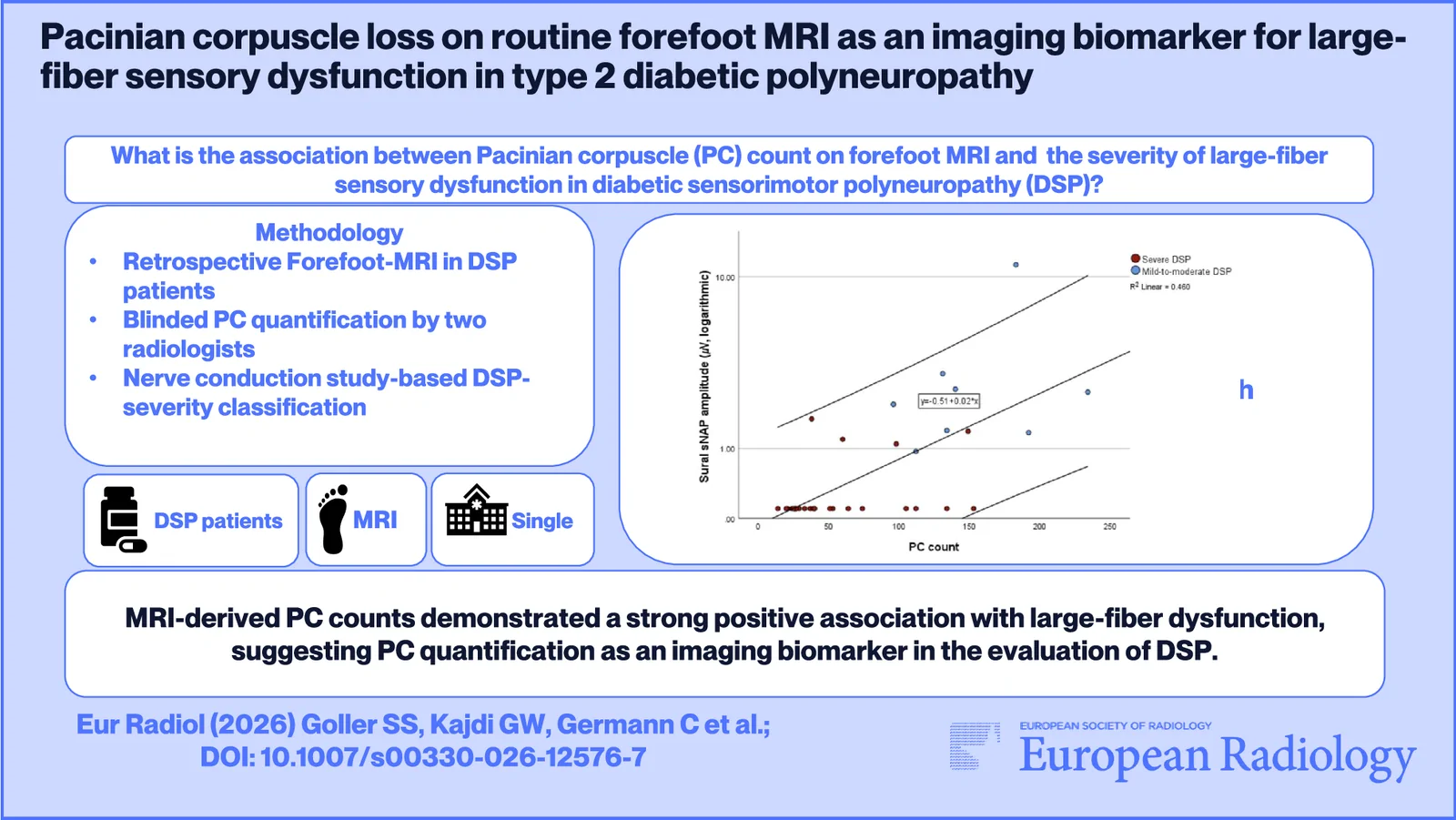

## Introduction

Pacinian corpuscles (PC), also known as Vater-Pacini or lamellar corpuscles, are mechanoreceptors that detect vibration and deep pressure stimuli [1]. These encapsulated sensory end organs are located at the dermal-hypodermal junction and in association with capsuloligamentous structures, with particularly high concentrations in the hands and feet [2]. PC are innervated by fast-conducting myelinated Aβ fibers and have a characteristic lamellar, onion-like appearance, due to their concentric arrangement of modified fibroblasts and fluid-filled spaces surrounding a central axon terminal [3].

Diabetic sensorimotor polyneuropathy (DSP) is one of the most common and disabling complications of type 2 diabetes, affecting up to 50% of patients during their disease course. About half of those affected report neuropathic pain, and many develop progressive sensory deficits, gait instability, and an increased risk of diabetic foot ulcers with subsequent infections that may lead to amputations [4–6]. DSP primarily involves the distal peripheral nerves, particularly large myelinated fibers such as Aβ fibers, resulting in reduced sensation to vibration, pressure, and proprioception [4–7]. Current diagnostic methods for DSP include clinical neurological assessments, quantitative sensory testing, nerve conduction studies (NCS), and skin biopsy to exclude other conditions, such as small fiber neuropathy [8–13]. While these techniques are well established, they can be time-consuming and invasive, require patient cooperation and specialized equipment, and may not be available in all clinical settings. Furthermore, NCS may fail due to signal loss in severe disease. Because Aβ fibers and related mechanoreceptors, such as PC, contribute to DSP’s pathophysiology, imaging-based evaluation of these structures could provide a new noninvasive tool for disease detection and monitoring. Building on recent advances in imaging that allow in vivo visualization of PC using high-resolution ultrasound and (ultra)-high-field MRI [14–17], we recently showed that PC can be reliably measured with clinical forefoot MRI, with the number and distribution of PC differing between patients with DSP and matched healthy controls [18]. These findings suggest that MRI-detectable PC loss may reflect underlying neuropathic processes. However, it remains unclear whether MRI-derived PC counts correlate with established electrophysiological parameters of large-fiber function. Specifically, it is unknown whether the number of PC visible on forefoot MRI correlates with DSP severity as assessed by NCS, which remains the gold standard for evaluating large-fiber impairment.

Therefore, this study aimed to examine whether MRI-derived PC counts in type 2-related DSP are associated with the severity of polyneuropathy and quantitative electrophysiological measures of sensory large-fiber function, thus assessing the potential of PC quantification as a promising imaging biomarker for DSP.

## Materials and methods

### Study design and participants

This retrospective single-center study was approved by the local institutional review board (Cantonal Ethics Committee Zurich) and conducted in accordance with national ethical standards and the principles of the Declaration of Helsinki and its subsequent amendments.

### Patients

**Fig. 1:**
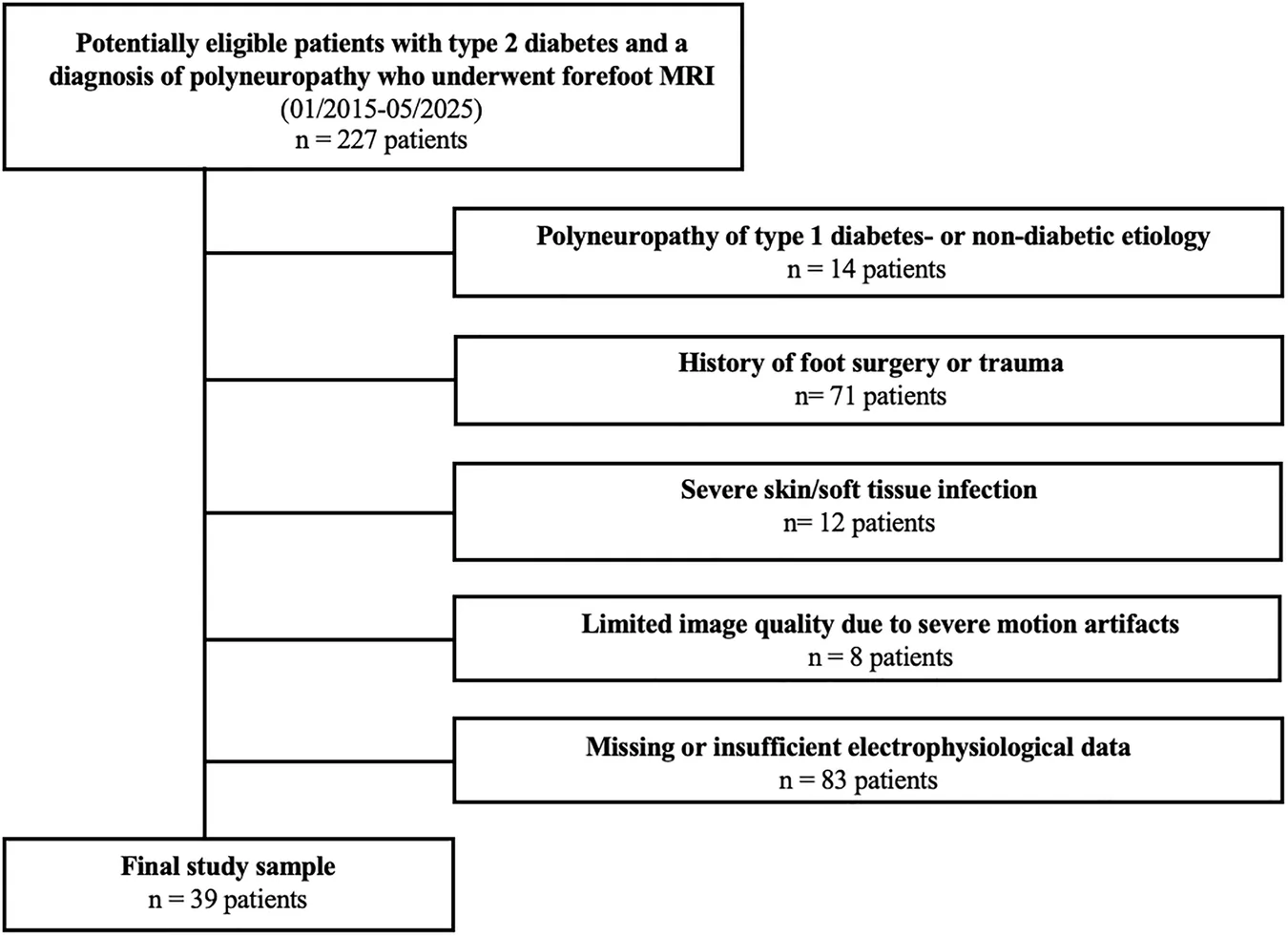
Flowchart illustrating the patient selection process. Out of the *n* = 227 potentially eligible patients with type 2 diabetes and polyneuropathy referred by the Neurology department and the Diabetic foot unit for clinical forefoot MRI, *n* = 188 were excluded. This resulted in a sample of *n* = 39 patients who met the study criteria

All patients included in this retrospective study provided written informed consent, authorizing the use of their health-related data for research purposes. We reviewed patients in the radiology information system (RIS) who were referred by the Neurology department and the Diabetic foot unit for clinically indicated forefoot MRI between January 2015 and May 2025. Patients qualified if they had a diagnosis of type 2 diabetes and a neurologically confirmed diagnosis of DSP according to current guidelines [19]. Additionally, all included patients underwent quantitative neurological assessment, including NCS. Patients with a diagnosis of type 1 diabetes, prior surgery or trauma, severe skin or soft tissue infections that caused pronounced edema preventing reliable assessment of PC counts, limited image quality due to motion artifacts, or insufficient NCS data were excluded. This resulted in 39 patients meeting the study criteria (Fig. 1). Of these, four had already been reported in our previous study [18]. Clinical data, including the duration of type 2 diabetes and the most recent Hemoglobin A1c (HbA1c) value, were extracted from patient charts.

### Imaging protocol

Patients underwent clinically indicated MRI of the forefoot, from the Lisfranc joint line to the toe tips, using 1.5 T (MAGNETOM Sola, MAGNETOM Avanto Fit, Siemens Healthineers) (*n* = 14) and 3 T units (MAGNETOM Vida, Siemens Healthineers) (*n* = 25) with a dedicated 16-channel foot coil. Clinical indications for MRI included suspected infection (*n* = 37), suspected insufficiency fracture (*n* = 1), and suspected Morton’s neuroma (*n* = 1). All patients were imaged in the supine position using a routine forefoot protocol that included coronal and sagittal fat-saturated (fs) proton-density (PD)-weighted, sagittal and transversal T1-weighted, and transversal fs and non-fs T2-weighted turbo spin-echo (TSE) sequences (detailed sequence parameters are provided in Supplementary Table 1).

### Image analysis

Image analysis was performed on a picture archiving and communication system (PACS) workstation (Merlin, Phoenix-PACS). All MRI studies were anonymized and independently reviewed by two musculoskeletal fellowship-trained radiologists (S.S.G. and G.W.K., each with six years of experience). Examinations were analyzed in a random order, and both readers were blinded to clinical data and to each other’s results.

PC assessment was conducted using the methodology from our previously published study [18]. PC numbers were evaluated separately for two distinct locations, referred to as *“subcutaneous”*, meaning PC located at the junction of the dermis and hypodermis in the subcutaneous fat, and *“deep”*, meaning PC located along the deep muscle fascia and adjacent to capsuloligamentous structures. Readers assessed the number of PC at these two locations for the following subregions for each digit: distal phalanx (I–V), middle phalanx (II–V), proximal phalanx (I–V), metatarsophalangeal (MTP) joint (I–V), and metatarsal bone (I–V).

PC were distinguished from blood vessels by their consistently hyperintense appearance, whereas the latter show a central hypointense area with a hyperintense rim on cross-section and linear branching across slices [14, 18]. Additionally, one radiologist (S.S.G.) evaluated the maximum PC diameter. PC were viewed in all three spatial planes and measured in the plane with the maximum diameter.

### NCS

Quantitative electrophysiological parameters, including compound muscle action potentials (cMAP), sensory nerve action potentials (sNAP), and corresponding nerve conduction velocities (NCV), were extracted from patient charts. All parameters were interpreted according to established reference standards as described in Vogel’s textbook of clinical neurophysiology [20], which provides normative data for the tibial, peroneal, and sural nerves. Amplitudes > 5 mV (motor) or > 5 µV (sensory) were considered within the normal range, values between 2 and 5 mV/µV were classified as reduced, and amplitudes < 2 mV/µV were defined as abolished. NCV was evaluated using age-adjusted reference limits [20] (Supplementary Table 2).

Based on NCS results, patients were classified into two polyneuropathy severity grades (mild-to-moderate and severe) using a pragmatic, empirically derived decision model developed to reflect routine clinical electrophysiological assessment. The classification was defined in consensus by two senior neurologists with extensive experience in NCS interpretation and was based on cMAP and sNAP amplitudes, as well as NCV of the tibial, peroneal, and sural nerves. Severe polyneuropathy was defined by abolished amplitudes (< 2 mV/µV) and pathological NCV affecting both motor (tibial, peroneal, or both) and sensory nerves. Mild-to-moderate polyneuropathy was assigned when amplitudes in at least two of the three tested nerves were normal (> 5 mV/µV) or reduced but recordable (2–5 mV/µV), with either normal or reduced NCV. No established ordinal severity grading system (e.g., the Baba classification) was applied, as a dichotomized model was considered more appropriate for the intended imaging correlation analysis and to reflect clinically meaningful disease stages.

Additionally, each case was categorized as motor-dominant, sensory-dominant, or mixed-type polyneuropathy according to the distribution of electrophysiological abnormalities. The sural sNAP amplitude was used as the primary electrophysiological parameter to assess the relationship between the PC count and quantitative NCS results (Spearman’s rank correlation analysis), as it is considered a sensitive and reliable indicator of large-fiber sensory dysfunction in DSP. It reflects degeneration of large myelinated Aβ fibers, which contribute to mechanosensory function and are anatomically and functionally linked to PC, thereby providing a physiologically meaningful structure-function comparison [21–23]. Abolished sural sNAP amplitudes were coded as 0.1 µV for statistical analysis.

### Statistical analysis

All statistical analyses were conducted using SPSS Statistics (v. 29, IBM Corporation). Quantile-quantile plots and the Shapiro-Wilk test were used to assess the normal distribution of continuous variables. Descriptive statistics summarized patient characteristics, the number of PC identified on forefoot MRI, and quantitative electrophysiological parameters. Unless otherwise noted, nominal variables were reported as frequencies and percentages, while continuous variables were presented as mean ± standard deviation (SD) or median and interquartile range (IQR), depending on their distribution. Group comparisons were performed using the Mann–Whitney *U*-test, and a *p*-value < 0.05 was considered statistically significant. To ensure consistency across analyses, all statistical evaluations—except for the inter- and intra-reader agreement—were based on the measurements obtained from Reader 1. An a priori power analysis using G*Power (v. 3.1) was performed to determine the required sample size to detect an upper-medium correlation (ρ = 0.45) between the PC count and sural sNAP amplitudes using Spearman’s rank correlation analysis, with 80% power at a two-sided α level of 0.05. This analysis indicated that at least 36 participants were required. Associations were visualized with scatterplots. Inter- and intra-reader agreement for PC counts was assessed using intraclass correlation coefficients (ICC). Intra-reader agreement was evaluated by repeated analysis of the same MRI datasets after a 6-week interval. All subregions were analyzed separately for subcutaneous and deep PC. A two-way random-effects model with absolute agreement was applied. Agreement was interpreted as follows [24]: 0.0 = poor, 0.01–0.20 = slight, 0.21–0.40 = fair, 0.41–0.60 = moderate, 0.61–0.80 = substantial, and > 0.80 = almost perfect.

## Results

### Patient characteristics

The study included 39 DSP patients (mean age 67.9 ± 13.5 years, 29 males). General demographic and clinical data of the patient cohort are summarized in Table 1.

**Table 1:** Patient characteristics

	All DSP patients (*n* = 39)	Mild-to-moderate DSP patients^*^ (*n* = 10)	Severe DSP patients^*^(*n* = 29)	*p*-value^**^
Male sex (%)	29 (74.4)	7 (70)	22 (56.4)	0.72
Age [years]	67.9 ± 13.5	69.7 ± 15.1	67.3 ± 13.2	0.55
Height [cm]	175.9 ± 9.8	178.2 ± 13.7	175.2 ± 8.2	0.50
Weight [kg]	91.5 ± 17.0	89.1 ± 21.1	92.3 ± 15.6	0.57
Body mass index [kg/m^2^]	27.2 ± 9.2	28.1 ± 6.4	26.9 ± 10.1	0.33
Forefoot MRI right side (%)	22 (56.4)	5 (50)	17 (58.6)	0.64
Diabetes duration [years]	10 (5–18)	8 (4–17)	11 (6–21)	0.39
HbA1c^***^ [% NGSP]	7.3 ± 1.5	7.5 ± 1.5	7.3 ± 1.6	0.37

Nominal data are presented as frequencies with percentages in parentheses. Continuous data are presented as mean ± SD or median with IQR in parentheses, depending on the distribution

*DSP* diabetic sensorimotor polyneuropathy, *HbA1c* hemoglobin A1c

^*^ Group classification based on the empirical decision model, which uses motor and sensory nerve amplitudes, as well as age-adjusted NCV

^**^ Group differences were evaluated using the Mann–Whitney *U*-test; the corresponding *p*-values are provided

^***^ HbA1c values were measured according to the National Glycohemoglobin Standardization Program (NGSP)

### Nerve conduction study (NCS) results and polyneuropathy severity grading

**Fig. 2:**
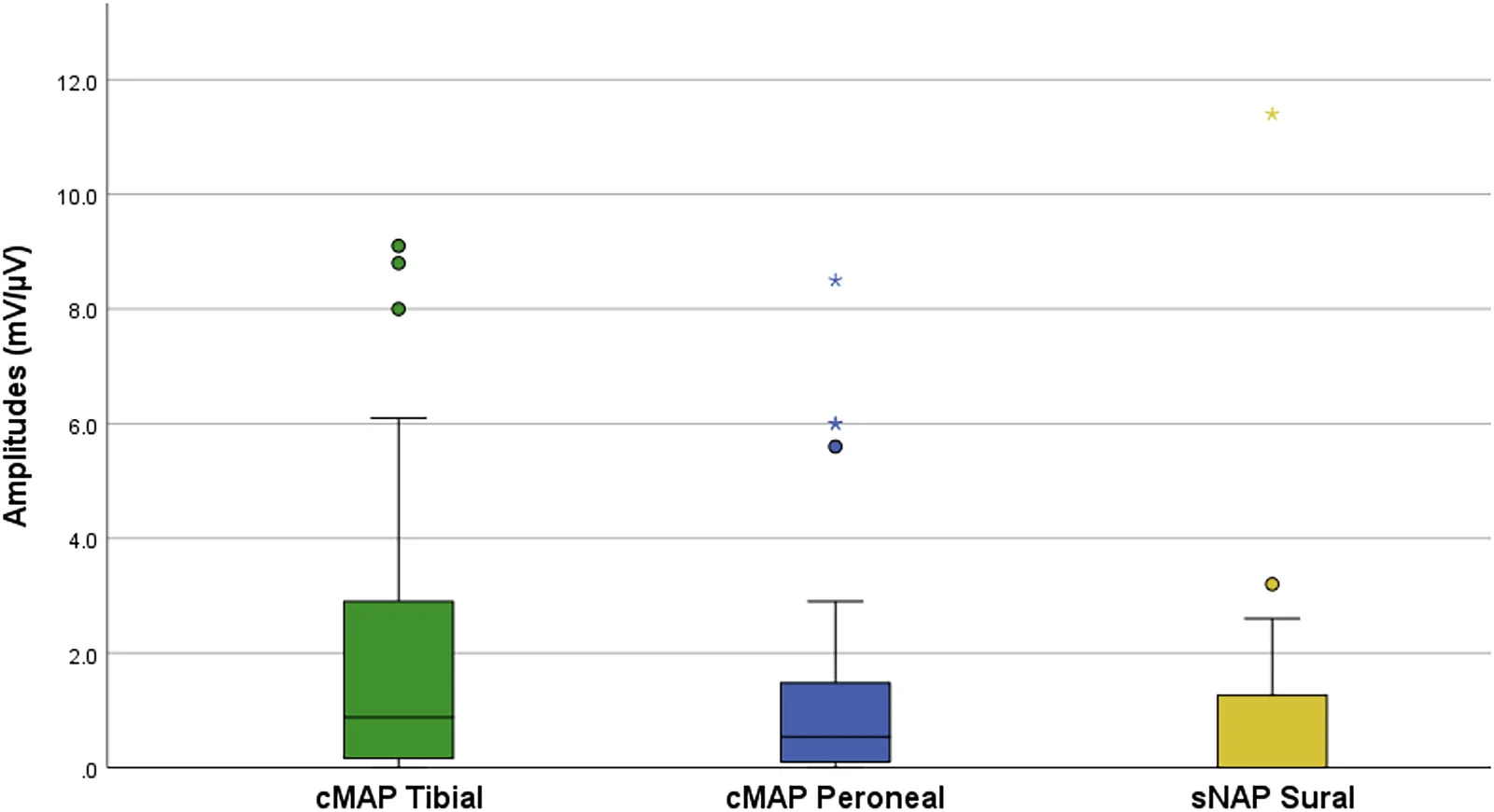
Boxplots showing the distribution of compound muscle action potential (cMAP) amplitudes for the tibial and peroneal nerves, as well as sensory nerve action potential (sNAP) amplitudes for the sural nerve

**Fig. 3:**
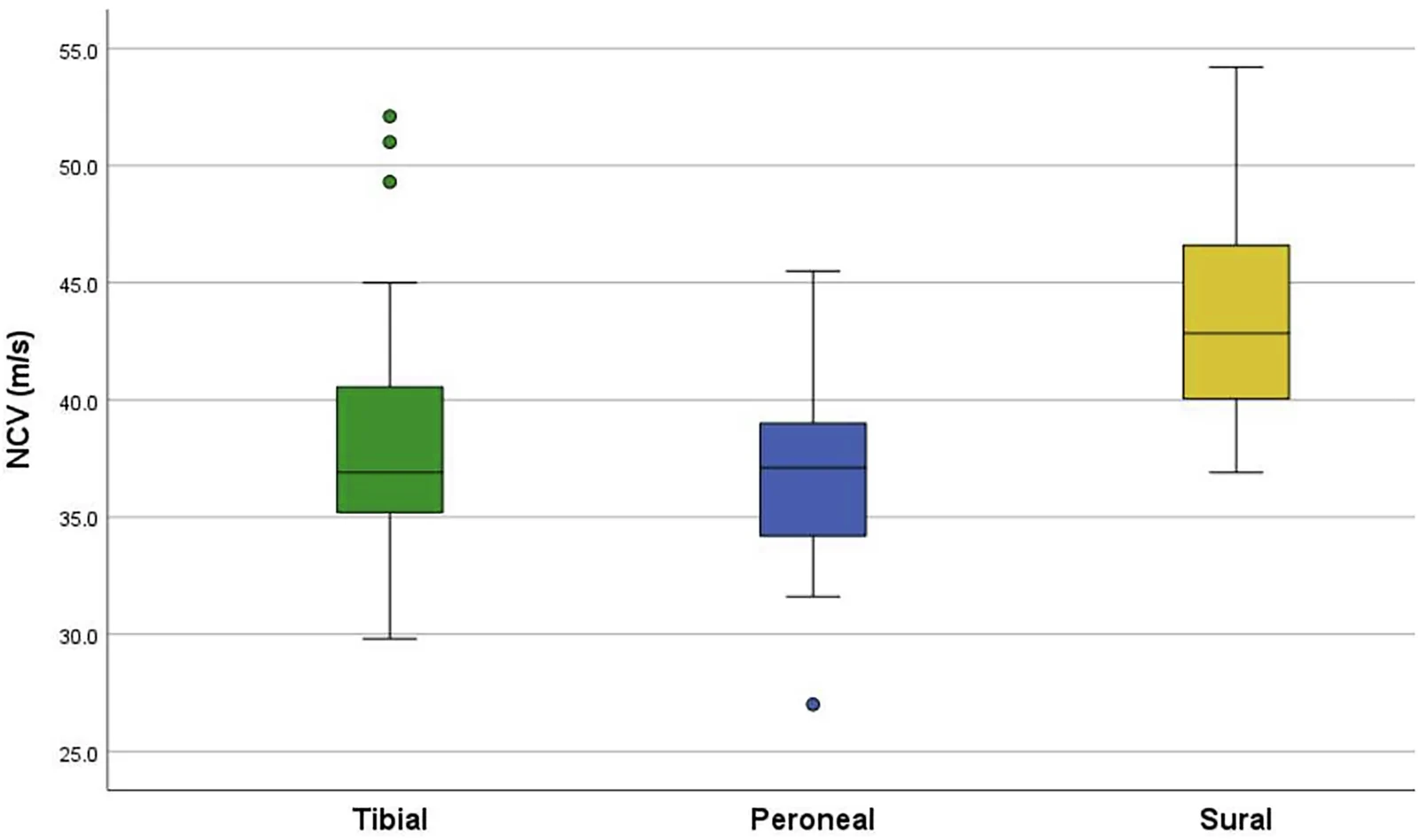
Boxplots illustrating the nerve conduction velocities (NCV) of the tibial, peroneal, and sural nerves

Patients underwent NCS within a median interval of five months before or after MRI. Nerve conduction data were available for 37 patients for the tibial nerve, 29 for the peroneal nerve, and 36 for the sural nerve. Based on established reference values [20], tibial nerve amplitudes were normal in four (10.8%), pathological in ten (27.0%), and abolished in 23 (62.2%) patients; peroneal nerve amplitudes were normal in four (13.8%), pathological in two (6.9%), and abolished in 23 (79.3%) patients; sural nerve amplitudes were normal in one (2.8%), pathological in four (11.1%), and abolished in 31 (86.1%) patients. Pathological NCV values were observed in 27 (69.2%) patients for the tibial nerve, 24 (82.8%) for the peroneal nerve, and 28 (77.8%) for the sural nerve, with the remaining values within age-adjusted reference ranges. Results are summarized in Table 2 (median, IQR) and illustrated in Figs. 2 and 3.

**Table 2:** Electrophysiological parameters

Parameter^*^	Nerve	Median (IQR)
cMAP [mV]	Tibial	0.9 (0.1–3.1)
NCV [m/s]	Tibial	36.9 (35.2–40.7)
cMAP [mV]	Peroneal	0.5 (0.1–1.5)
NCV [m/s]	Peroneal	37.1 (34.2–39.1)
sNAP [µV]	Sural	0 (0–1.3)
NCV [m/s]	Sural	42.9 (39.1–47.7)

Continuous data are presented as median with IQR in parentheses

*cMAP* compound muscle action potential, *IQR* interquartile range, *NCV* nerve conduction velocity, *sNAP* sensory nerve action potential

^*^ Reference normative values were adapted from Vogel [20]: amplitudes > 5 mV/µV are considered normal, 2–5 mV/µV pathological, and < 2 mV/µV abolished. Normative values for nerve conduction velocities are adjusted for age

Based on the empirical decision model, ten out of 39 patients (25.6%) were classified as having mild-to-moderate polyneuropathy, including three with sensory-dominant and seven with mixed-type sensorimotor polyneuropathy. The remaining 29 patients (74.4%) were categorized as having severe polyneuropathy, all of whom had mixed-type sensorimotor polyneuropathy. None of the patients had motor-dominant polyneuropathy. There were no statistically significant differences between patients with mild-to-moderate and those with severe polyneuropathy in key demographic and clinical characteristics, including diabetes duration and HbA1c levels (see Table 1 for details, Mann–Whitney *U*-test, all *p* ≥ 0.33).

### PC number, distribution, and size on forefoot MRI

**Fig. 4:**
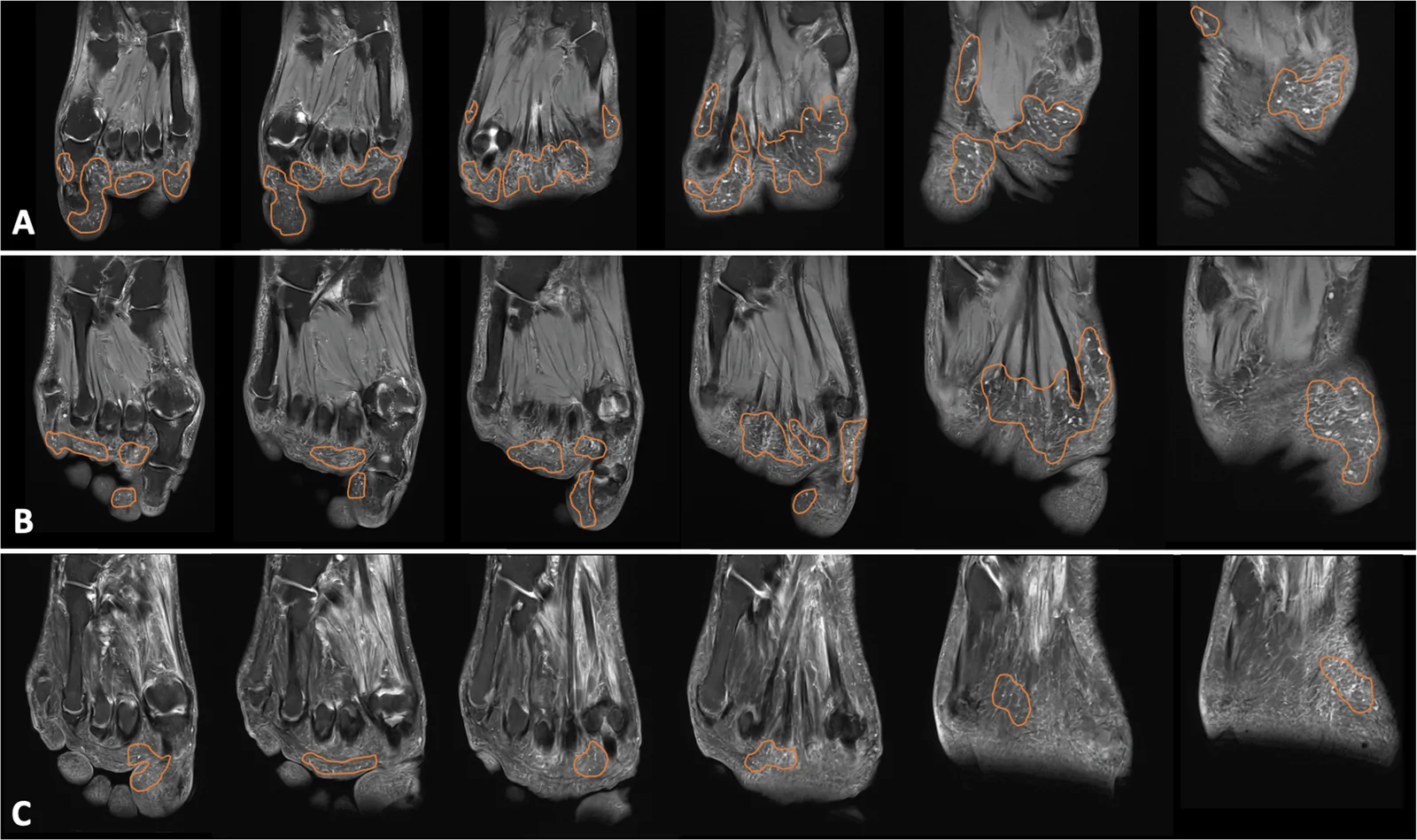
Example consecutive coronal fs PD-weighted TSE images of the forefoot showing (**A**) a 66-year-old male without DSP, who underwent imaging to evaluate progression of Lisfranc joint osteoarthritis (left foot; not part of the current study, but shown here for didactic purposes). **B** a 65-year-old female (right foot) with mild-to-moderate DSP, and **C** a 77-year-old male (right foot) with severe DSP, illustrate a progressive reduction in the number of identifiable PC. Rows **A**–**C** are shown in the same anatomical orientation, allowing direct comparison of findings across subjects. Contour markings schematically highlight areas of PC to aid visualization

The total number of PC in the forefoot of patients with severe polyneuropathy was significantly lower than in those with mild-to-moderate polyneuropathy (mean ± SD [range]: 54.1 ± 40.6 [14–153] vs 146.1 ± 43.2 [96–234]; *p* < 0.001) (Fig. 4).

Additionally, this difference was also evident across individual subregions of each digit (*p* ≤ 0.028) (Table 3). Both patients with mild-to-moderate and those with severe polyneuropathy showed a disrupted, “spot-like” PC distribution pattern. Furthermore, there was no apparent anatomical clustering of PC, nor any specific preference for particular subregions, such as the phalanges, MTP joints, or metatarsal bones. In both groups, the maximum PC diameter was 3 mm (range 1-3 mm).

**Table 3:** Count of PC in different subregions for each digit I–V

		All DSP patients (*n* = 39)	Mild-to-moderate DSP patients (*n* = 10)	Severe DSP patients (*n* = 29)	*p*-value^1^
Digit I	Distal phalanx				
	Overall	4 ± 2.8 (0–13)	6.8 ± 3.0 (3–13)	2.6 ± 2.2 (0–10)	< 0.001
	Subcutaneous	1.8 ± 1.6 (0–6)	3.2 ± 1.3 (2–6)	1.2 ± 1.3 (0–5)	< 0.001
	Deep	2 ± 1.8 (0–7)	3.6 ± 1.9 (1–7)	1.4 ± 1.5 (0–6)	0.002
	Proximal phalanx				
	Overall	4 ± 3.9 (0–13)	8 ± 2.9 (4–13)	2.7 ± 3.2 (0–12)	< 0.001
	Subcutaneous	1.8 ± 1.8 (0–6)	3.6 ± 1.3 (2–6)	1.2 ± 1.6 (0–6)	< 0.001
	Deep	2.2 ± 2.2 (0–7)	4.2 ± 1.7 (2–7)	1.4 ± 1.7 (0–7)	< 0.001
	MTP joint				
	Overall	3.8 ± 3.8 (0–15)	8.7 ± 3.1 (5–15)	2.1 ± 2.3 (0–10)	< 0.001
	Subcutaneous	1.9 ± 2.0 (0–8)	4.5 ± 2.0 (2–8)	1 ± 1.1 (0–4)	< 0.001
	Deep	1.9 ± 1.9 (0–7)	4.2 ± 1.3 (2–7)	1.1 ± 1.4 (0–6)	< 0.001
	Metatarsal bone				
	Overall	4.1 ± 3.7 (0–14)	8.6 ± 3.0 (0–14)	2.6 ± 2.5 (0–10)	< 0.001
	Subcutaneous	2.4 ± 2.0 (0–8)	5 ± 1.7 (3–8)	1.4 ± 1.2 (0–4)	< 0.001
	Deep	1.8 ± 1.8 (0–6)	3.6 ± 1.3 (2–6)	1.1 ± 1.4 (0–6)	< 0.001
Digit II	Distal phalanx				
	Overall	3.2 ± 2.4 (0–7)	5.7 ± 1.3 (3–7)	2.3 ± 2.0 (0–7)	< 0.001
	Subcutaneous	2 ± 1.6 (0–5)	3.9 ± 0.9 (2–5)	1.4 ± 1.3 (0–5)	< 0.001
	Deep	1.2 ± 1.0 (0–4)	1.8 ± 0.6 (1–3)	0.9 ± 1.0 (0–4)	0.006
	Middle phalanx				
	Overall	2.5 ± 2.1 (0–8)	4.9 ± 1.7 (3–8)	1.7 ± 1.4 (0–6)	< 0.001
	Subcutaneous	1.3 ± 1.1 (0–4)	2.5 ± 0.7 (2–4)	0.9 ± 0.9 (0–4)	< 0.001
	Deep	1.2 ± 1.1 (0–4)	2.4 ± 1.1 (1–4)	0.8 ± 0.7 (0–2)	< 0.001
	Proximal phalanx				
	Overall	3.3 ± 3.2 (0–11)	6.5 ± 2.8 (3–11)	0.8 ± 2.5 (0–9)	< 0.001
	Subcutaneous	2 ± 2.0 (0–7)	4 ± 1.8 (2–7)	1.3 ± 1.6 (0–5)	< 0.001
	Deep	1.3 ± 1.3 (0–4)	2.5 ± 1.3 (1–4)	0.9 ± 1.1 (0–4)	< 0.001
	MTP joint				
	Overall	2.1 ± 0.7 (0–7)	4.1 ± 1.5 (3–7)	1.3 ± 1.0 (0–4)	< 0.001
	Subcutaneous	1.2 ± 1.0 (0–4)	2.5 ± 0.8 (1–4)	0.8 ± 0.7 (0–3)	< 0.001
	Deep	0.9 ± 0.9 (0–3)	1.8 ± 0.8 (1–3)	0.6 ± 0.6 (0–2)	< 0.001
	Metatarsal bone				
	Overall	3.2 ± 3.0 (0–12)	7.1 ± 2.5 (4–12)	1.9 ± 1.7 (0–7)	< 0.001
	Subcutaneous	2 ± 1.9 (0–8)	4.3 ± 1.9 (2–8)	1.2 ± 1.0 (0–4)	< 0.001
	Deep	1.2 ± 1.2 (0–4)	2.8 ± 0.9 (1–4)	0.7 ± 0.7 (0–3)	< 0.001
Digit III	Distal phalanx				
	Overall	3.7 ± 3.0 (0–11)	6.3 ± 2.6 (2–11)	2.9 ± 2.6 (0–11)	< 0.001
	Subcutaneous	2.5 ± 1.8 (0–7)	4 ± 1.6 (2–7)	2 ± 1.7 (0–7)	0.002
	Deep	1.2 ± 1.2 (0–4)	2.3 ± 1.2 (0–4)	0.9 ± 1.0 (0–4)	0.002
	Middle phalanx				
	Overall	2.7 ± 2.3 (0–8)	5 ± 1.9 (2–8)	2 ± 1.9 (0–7)	< 0.001
	Subcutaneous	1.5 ± 1.3 (0–4)	2.7 ± 1.2 (1–4)	1.0 ± 1.1 (0–4)	< 0.001
	Deep	1.3 ± 1.2 (0–4)	2.3 ± 1.2 (1–4)	0.9 ± 1.0 (0–3)	0.003
	Proximal phalanx				
	Overall	3.2 ± 2.6 (0–9)	5.7 ± 1.9 (3–9)	2.3 ± 2.3 (0–8)	< 0.001
	Subcutaneous	1.9 ± 1.7 (0–5)	3.4 ± 1.2 (2–5)	1.3 ± 1.5 (0–5)	< 0.001
	Deep	1.3 ± 1.1 (0–4)	2.3 ± 0.9 (1–4)	0.9 ± 0.9 (0–3)	< 0.001
	MTP joint				
	Overall	2.5 ± 2.1 (0–7)	4.7 ± 2.0 (2–7)	1.7 ± 1.6 (0–6)	< 0.001
	Subcutaneous	1.4 ± 1.3 (0–4)	2.7 ± 0.9 (1–4)	1 ± 1.1 (0–4)	< 0.001
	Deep	1 ± 1.0 (0–4)	2 ± 1.2 (0–4)	0.7 ± 0.7 (0–2)	0.003
	Metatarsal bone				
	Overall	3.8 ± 2.6 (0–11)	6.3 ± 2.5 (3–11)	3 ± 2.1 (0–9)	< 0.001
	Subcutaneous	2.4 ± 1.7 (0–7)	3.9 ± 1.7 (2–7)	1.9 ± 1.5 (0–6)	0.001
	Deep	1.5 ± 1.0 (0–4)	2.4 ± 1.0 (1–4)	1.1 ± 0.8 (0–3)	0.001
Digit IV	Distal phalanx				
	Overall	3.8 ± 2.5 (0–9)	5.6 ± 2.5 (1–9)	3.1 ± 2.2 (0–8)	0.007
	Subcutaneous	2.2 ± 1.7 (0–6)	3.5 ± 1.6 (1–6)	1.8 ± 1.5 (0–6)	0.006
	Deep	1.5 ± 0.9 (0–3)	2.1 ± 1.0 (0–3)	1.3 ± 0.8 (0–3)	0.028
	Middle phalanx				
	Overall	2.4 ± 1.9 (0–8)	4.6 ± 1.5 (3–8)	1.6 ± 1.4 (0–4)	< 0.001
	Subcutaneous	1.3 ± 1.1 (0–4)	2.3 ± 0.8 (1–4)	1 ± 0.9 (0–3)	< 0.001
	Deep	1.1 ± 1.1 (0–4)	2.3 ± 0.9 (1–4)	0.6 ± 0.8 (0–2)	< 0.001
	Proximal phalanx				
	Overall	3.3 ± 3.2 (0–12)	6.7 ± 2.8 (2–12)	2.1 ± 2.4 (0–8)	< 0.001
	Subcutaneous	1.9 ± 1.9 (0–7)	3.6 ± 1.8 (1–7)	1.2 ± 1.5 (0–6)	< 0.001
	Deep	1.4 ± 1.5 (0–5)	3.1 ± 1.2 (1–5)	0.9 ± 1.1 (0–4)	< 0.001
	MTP joint				
	Overall	2.7 ± 2.0 (0–7)	4.7 ± 2.0 (1–7)	2.1 ± 1.6 (0–5)	0.001
	Subcutaneous	1.6 ± 1.3 (0–4)	2.6 ± 1.3 (0–4)	1.2 ± 1.1 (0–3)	0.007
	Deep	1.2 ± 0.9 (0–3)	2.1 ± 0.7 (1–3)	0.8 ± 0.8 (0–2)	< 0.001
	Metatarsal bone				
	Overall	4.3 ± 3.3 (0–13)	8 ± 2.8 (3–13)	3 ± 2.3 (0–9)	< 0.001
	Subcutaneous	2.5 ± 2.1 (0–8)	4.9 ± 1.8 (2–8)	1.7 ± 1.5 (0–5)	< 0.001
	Deep	1.7 ± 1.3 (0–5)	3.1 ± 1.2 (1–5)	1.2 ± 1.0 (0–4)	< 0.001
Digit V	Distal phalanx				
	Overall	3.5 ± 2.6 (0–9)	6.1 ± 2.0 (3–9)	2.7 ± 2.2 (0–8)	< 0.001
	Subcutaneous	2.2 ± 1.8 (0–6)	3.9 ± 1.4 (2–6)	1.7 ± 1.6 (0–6)	< 0.001
	Deep	1.3 ± 0.9 (0–3)	2.2 ± 0.6 (1–3)	1 ± 0.8 (0–3)	< 0.001
	Middle phalanx				
	Overall	2.2 ± 1.5 (0–5)	3.8 ± 1.0 (2–5)	1.7 ± 1.3 (0–5)	< 0.001
	Subcutaneous	1.3 ± 1.0 (0–3)	2.2 ± 0.8 (1–3)	1 ± 0.9 (0–3)	0.002
	Deep	0.9 ± 0.8 (0–2)	1.6 ± 0.5 (1–2)	0.7 ± 0.7 (0–2)	0.001
	Proximal phalanx				
	Overall	2.9 ± 2.7 (0–9)	6 ± 2.1 (3–9)	2.9 ± 2.7 (0–9)	< 0.001
	Subcutaneous	1.3 ± 1.3 (0–4)	2.9 ± 0.9 (2–4)	0.8 ± 1.0 (0–4)	< 0.001
	Deep	1.6 ± 1.5 (0–5)	3.1 ± 1.4 (1–5)	1.0 ± 1.1 (0–3)	< 0.001
	MTP joint				
	Overall	2.8 ± 2.3 (0–8)	5.8 ± 1.7 (3–8)	1.8 ± 1.5 (0–6)	< 0.001
	Subcutaneous	1.8 ± 1.6 (0–5)	3.6 ± 1.2 (2–5)	1.2 ± 1.1 (0–4)	< 0.001
	Deep	1 ± 1.0 (0–3)	2.2 ± 0.8 (1–3)	0.6 ± 0.6 (0–2)	< 0.001
	Metatarsal bone				
	Overall	3.8 ± 2.8 (0–10)	6.2 ± 2.4 (3–10)	2.9 ± 2.4 (0–9)	< 0.001
	Subcutaneous	2.1 ± 1.6 (0–5)	3.2 ± 1.3 (1–5)	1.7 ± 1.5 (0–5)	0.006
	Deep	1.7 ± 1.4 (0–5)	3 ± 1.3 (1–5)	1.2 ± 1.1 (0–4)	< 0.001

The table shows the number of PC on the forefoot MRI. PC counts were not normally distributed; values are presented as mean ± SD (range) for descriptive purposes. A subgroup analysis was conducted to compare patients with mild-to-moderate and severe polyneuropathy. Group differences were evaluated using the Mann–Whitney *U*-test; the corresponding *p*-values are provided

*DSP* diabetic sensorimotor polyneuropathy, *MTP* metatarsophalangeal (joint)

### Correlation between PC count and sural sNAP amplitudes

**Fig. 5:**
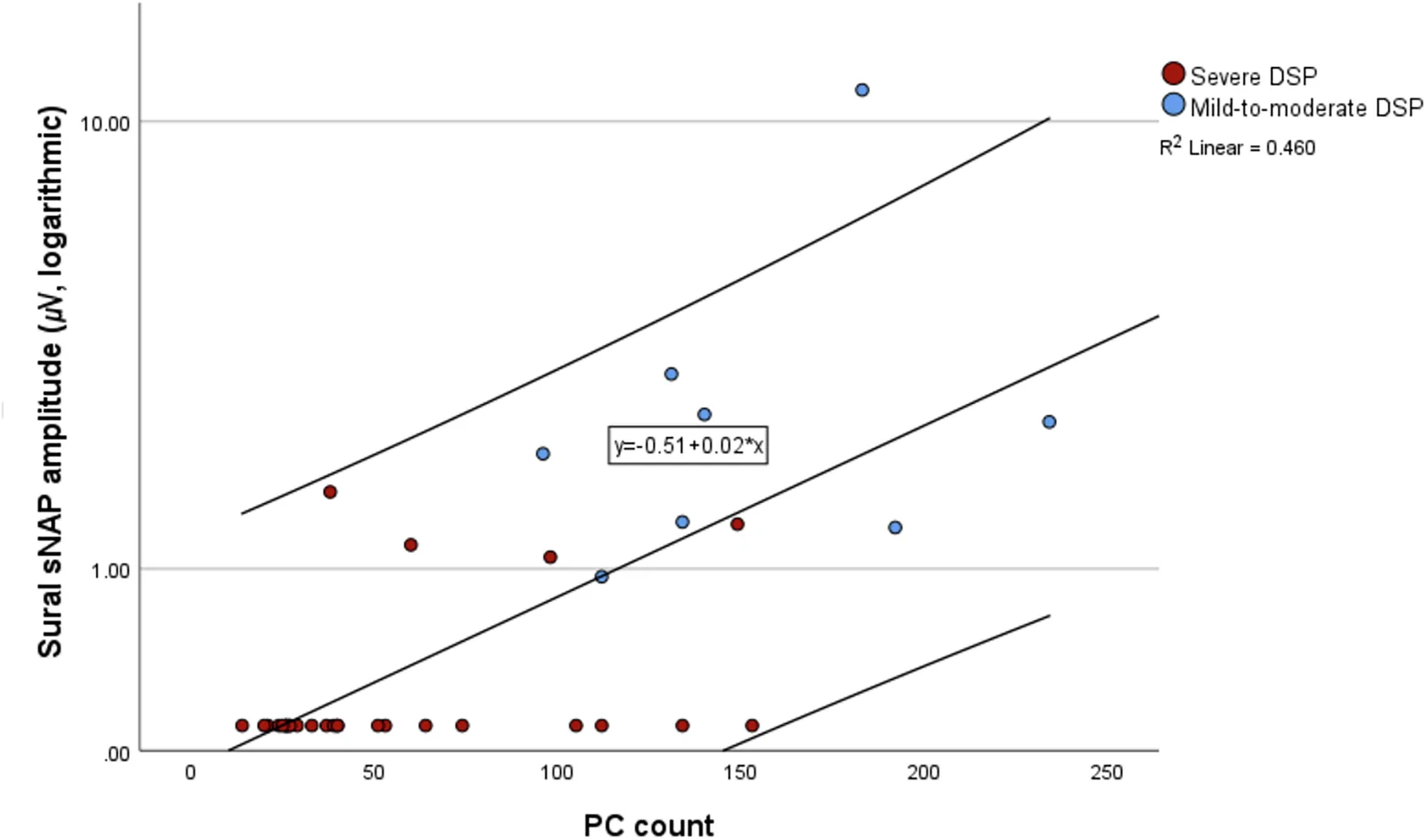
Scatterplot of total PC counts vs sural sensory nerve action potential (sNAP) amplitudes in patients with type 2 DSP. Points are color-coded by severity (red = severe DSP, blue = mild-to-moderate DSP). The *Y*-axis is logarithmic to improve visualization of low sNAP values. Spearman’s rank correlation analysis reveals a strong, positive relationship between total PC counts and sural sNAP amplitudes (ρ = 0.638, *p* < 0.001). A linear regression line fitted on log-transformed sNAP amplitudes is shown (*R*² = 0.46), with the 95% CI indicated

Spearman’s rank correlation analysis revealed a strong, positive relationship between total PC counts and sural sNAP amplitudes (ρ = 0.638, *p* < 0.001), indicating that patients with higher MRI-derived PC counts tend to have better preserved large-fiber sensory function. To illustrate this relationship, sural sNAP amplitudes were plotted on a logarithmic scale, with a linear regression line fitted on the log-transformed data (*R*² = 0.46, indicating that 46% of the variance in amplitudes is explained by PC counts; 95% confidence interval (CI) shown in Fig. 5). This pattern remained consistent when patients were grouped by polyneuropathy severity: those with severe DSP exhibited significantly lower or absent sural sNAP amplitudes (median [range]: 0.1 [0.1–1.7] µV) and lower PC counts, whereas patients with mild-to-moderate DSP had higher sNAP amplitudes (median [range]: 2.3 [0.9–11.4] µV) along with higher PC counts. These differences were statistically significant (*p* < 0.001), further supporting the hypothesis that MRI-detectable PC loss closely reflects functional impairment of large myelinated fibers in DSP.

### Inter- and intra-reader agreement

The inter-reader agreement ranged from substantial to almost perfect for assessing PC numbers (ICC range: 0.61–0.94), depending on the anatomical sub-region. The intra-reader agreement for both readers also ranged from substantial to almost perfect (ICC range for Reader 1: 0.64–0.95, ICC range for Reader 2: 0.66–0.96). Detailed ICC values, including 95% CI, are provided in Supplementary Tables 3–5.

## Discussion

This study provides the first evidence that the PC count on routine forefoot MRI is directly related to the severity of DSP, as assessed by NCS.

While previous research has shown that PC counts are markedly reduced in patients with DSP compared to healthy individuals [18], the present work demonstrates that MRI-based PC counts are not only diminished in DSP but also quantitatively reflect the degree of large-fiber sensory dysfunction. The significant difference in PC counts between mild-to-moderate and severe DSP, consistently observed across all anatomical subregions, highlights a structural imaging marker that has not previously been shown to indicate clinically relevant disease severity. Furthermore, the finding that even patients with mild-to-moderate DSP exhibit early PC loss underscores the potential of PC imaging to detect subtle quantitative abnormalities that may not yet be apparent through clinical examination. These results extend prior histopathological observations [25], confirming in vivo that PC reduction parallels clinical neuropathy severity and can be visualized on routine MRI.

A key finding of this study is the strong, positive relationship between MRI-derived PC counts and sural sNAP amplitudes, which are a reliable electrophysiological indicator of large-fiber sensory function. Patients with severe DSP exhibited significantly reduced or absent sural sNAP amplitudes and markedly lower PC counts, whereas those with mild-to-moderate DSP maintained both higher sNAP amplitudes and PC counts. This structural-functional link supports the biological plausibility that MRI-visible changes in PC counts reflect the extent of peripheral nerve damage. Mechanistically, the selective vulnerability of mechanoreceptors to metabolic and microvascular injury may explain the observed PC loss, highlighting a structural correlate of large-fiber degeneration in DSP [5, 25, 26]. The consistency of this relationship across severity levels further suggests that PC measurement can not only differentiate between different disease stages but also potentially reflect a continuous spectrum of large-fiber nerve dysfunction.

The potential clinical implications are significant. Forefoot MRI is routinely used in diabetic patients to evaluate infections, ulcers, or other musculoskeletal issues. PC quantification can be obtained from standard clinical sequences without increasing scan time, providing a noninvasive method that could complement NCS. This approach could be particularly beneficial for patients unable to tolerate NCS, in settings with limited neurophysiology services, or in cases of severe polyneuropathy where nerve amplitudes are undetectable. The reliability of PC quantification in this study was high, with both inter- and intra-reader agreement ranging from substantial to almost perfect across anatomical subregions. This suggests that the method is suitable for wider clinical and research applications. Prospective longitudinal studies could evaluate whether PC quantification detects early subclinical neuropathy, predicts DSP progression, or monitors treatment response.

Several limitations should be acknowledged. First, the retrospective design may introduce selection bias. Second, sural sNAP amplitudes were unavailable for a small subset (*n* = 3), but the final sample was adequate for correlation analysis. Third, a healthy control group was not included, but normal PC patterns in the forefoot have been well documented previously [18]. Finally, the regression analysis revealed that 46% of the variance in sural sNAP amplitudes was explained by PC counts, indicating that PC loss is strongly associated with large-fiber dysfunction. However, other factors, such as diabetes duration, glycemic control, microvascular changes, and genetic susceptibility, probably also contribute to variability in sensory impairment. Our sample size, however, was too small to reliably assess the relative influence of these variables in a multivariate regression model. Additionally, the relatively small number of patients with mild-to-moderate polyneuropathy may limit the statistical power for subgroup analyses and affect the generalizability of our findings. Future studies with larger cohorts are needed to clarify how these factors interact and to further validate MRI-based PC loss as a marker of large-fiber dysfunction in DSP.

In conclusion, MRI-based quantification of PC in the forefoot closely reflects large-fiber sensory dysfunction in type 2 DSP. These findings highlight the potential of the PC count as a noninvasive imaging biomarker of polyneuropathy severity and encourage further longitudinal research to evaluate its diagnostic and prognostic value in clinical practice.

## Supplementary information

Supplementary information

## Abbreviations

CI: Confidence interval
cMAP: Compound muscle action potential
DSP: Diabetic sensorimotor polyneuropathy
fs: Fat-saturated
HbA1c: Hemoglobin A1c
ICC: Intraclass correlation coefficient
IQR: Interquartile range
MTP: Metatarsophalangeal (joint)
NCS: Nerve conduction study
NCV: Nerve conduction velocity
PACS: Picture archiving and communication system
PC: Pacinian corpuscle
PD: Proton-density
sNAP: Sensory nerve action potential
SD: Standard deviation
TSE: Turbo spin-echo
