# Supplementary material for: Pacinian corpuscle loss on routine forefoot MRI as an imaging biomarker for large-fiber sensory dysfunction in type 2 diabetic polyneuropathy
Source: Eur Radiol. 2026 Apr 29;36(9):7020–31. doi: 10.1007/s00330-026-12576-7 (PMC13710575; doi:10.1007/s00330-026-12576-7)
Supplement: Supplementary file 1 [file 330_2026_12576_MOESM1_ESM.pdf]

Pacinian Corpuscle Loss on Routine Forefoot MRI as an Imaging Biomarker for Large-Fiber Sensory Dysfunction in Type 2 Diabetic Polyneuropathy
ELECTRONIC SUPPLEMENTARY MATERIAL

Supplementary Material

Supplementary Table 1: Forefoot MRI protocol.

Sequence *	Cor PD fs TSE	Sag PD fs TSE	Sag T1 TSE	Tra T1 TSE	Tra T2 TSE	Tra T2 fs TSE
Echo time [ms]	32	33	12	11	71	67
Repetition time [ms]	3500	4510	680	700	4500	5000
Bandwidth [Hz/px]	302	302	266	260	248	246
Acquisition matrix	448x347	448x246	448x246	400x288	448x325	384x278
Slice thickness [mm]	3	3	3	3	3	3
Slice number	20	34	34	24	24	24
FOV [mm]	158x163	160x110	159x110	117x130	117x130	117x130
Acquisition time [min:sec]	1:20	1:34	0:53	0:36	0:49	2:05

Cor, coronal; FOV, field of view; fs, fat-saturated; Hz, Hertz; PD, proton-density; px, pixel; sag, sagittal; tra, transversal; TSE, turbo spin-echo.

** A subset of scans was acquired using the current standard protocol for the 3T MRI scanners. For scans performed earlier or on other MRI systems, slight deviations in acquisition parameters may apply.*

Supplementary Table 2: Age-dependent normative reference ranges for nerve conduction velocity of tibial, peroneal, and sural nerves based on Vogel [20]

Age [years]	Tibial NCV [m/s]	Peroneal NCV [m/s]	Sural NCV [m/s]
20	43	45	46
30	42	44	45
40	41	43	44
50	40	42	43
60	39	41	42
70	38	40	41

NCV, nerve conduction velocity

Supplementary Table 3: Inter-reader agreement.

			ICC *	Lower 95% CL	Upper 95% CL
**Digit I**	Distal phalanx	*Subcutaneous*	.745	.486	.870
		Deep	.857	.727	.925
	Proximal phalanx	*Subcutaneous*	.858	.722	.927
		Deep	.917	.841	.956
	MTP joint	*Subcutaneous*	.860	.735	.926
		Deep	.844	.671	.923
	Metatarsal bone	*Subcutaneous*	.842	.700	.917
		Deep	.766	.540	.879
**Digit II**	Distal phalanx	*Subcutaneous*	.785	.594	.887
		Deep	.823	.625	.912
	Middle phalanx	*Subcutaneous*	.656	.355	.818
		Deep	.630	.280	.808
	Proximal phalanx	*Subcutaneous*	.876	.763	.935
		Deep	.814	.602	.908
	MTP joint	*Subcutaneous*	.707	.443	.846
		Deep	.702	.436	.843
	Metatarsal bone	*Subcutaneous*	.853	.718	.923
		Deep	.827	.667	.910
**Digit III**	Distal phalanx	*Subcutaneous*	.881	.752	.941
		Deep	.779	.580	.884
	Middle phalanx	*Subcutaneous*	.881	.764	.939
		Deep	.657	.347	.820
	Proximal phalanx	*Subcutaneous*	.846	.705	.919
		Deep	.845	.704	.919
	MTP joint	*Subcutaneous*	.807	.631	.899
		Deep	.667	.324	.831
	Metatarsal bone	*Subcutaneous*	.891	.781	.945
		Deep	.767	.559	.877
**Digit IV**	Distal phalanx	*Subcutaneous*	.919	.845	.957
		Deep	.665	.370	.823
	Middle phalanx	*Subcutaneous*	.704	.437	.844
		Deep	.767	.559	.878
	Proximal phalanx	*Subcutaneous*	.864	.739	.929
		Deep	.854	.724	.923
	MTP joint	*Subcutaneous*	.758	.536	.873
		Deep	.657	.353	.819
	Metatarsal bone	*Subcutaneous*	.939	.884	.968
		Deep	.859	.728	.926
**Digit V**	Distal phalanx	*Subcutaneous*	.885	.780	.940
		Deep	.658	.272	.831
	Middle phalanx	*Subcutaneous*	.824	.667	.908
		Deep	.670	.154	.853
	Proximal phalanx	*Subcutaneous*	.606	.250	.793
		Deep	.799	.621	.894
	MTP joint	*Subcutaneous*	.880	.771	.937
		Deep	.810	.638	.900
	Metatarsal bone	*Subcutaneous*	.895	.800	.945
		Deep	.900	.808	.947

* The level of agreement was categorized as follows: 0.0 = poor, 0.01-0.20 = slight, 0.21-0.40 = fair, 0.41-0.60 = moderate, 0.61-0.80 = substantial, 0.81-1.00 = almost perfect agreement.

CL, confidence limit; ICC, intraclass correlation coefficient; MTP, metatarsophalangeal (joint).

Supplementary Table 4: Intra-reader agreement Reader 1.

			ICC *	Lower 95% CL	Upper 95% CL
**Digit I**	Distal phalanx	*Subcutaneous*	.805	.533	.909
		Deep	.846	.693	.921
	Proximal phalanx	*Subcutaneous*	.885	.759	.943
		Deep	.937	.841	.971
	MTP joint	*Subcutaneous*	.913	.835	.955
		Deep	.888	.781	.942
	Metatarsal bone	*Subcutaneous*	.867	.746	.930
		Deep	.800	.614	.896
**Digit II**	Distal phalanx	*Subcutaneous*	.811	.643	.901
		Deep	.676	.281	.844
	Middle phalanx	*Subcutaneous*	.644	.330	.812
		Deep	.764	.554	.876
	Proximal phalanx	*Subcutaneous*	.897	.804	.946
		Deep	.783	.513	.895
	MTP joint	*Subcutaneous*	.645	.327	.813
		Deep	.679	.269	.847
	Metatarsal bone	*Subcutaneous*	.924	.854	.960
		Deep	.823	.664	.907
**Digit III**	Distal phalanx	*Subcutaneous*	.912	.830	.954
		Deep	.862	.713	.930
	Middle phalanx	*Subcutaneous*	.908	.824	.952
		Deep	.853	.719	.923
	Proximal phalanx	*Subcutaneous*	.918	.843	.957
		Deep	.917	.843	.957
	MTP joint	*Subcutaneous*	.878	.767	.936
		Deep	.789	.491	.901
	Metatarsal bone	*Subcutaneous*	.927	.854	.963
		Deep	.822	.662	.906
**Digit IV**	Distal phalanx	*Subcutaneous*	.951	.906	.974
		Deep	.802	.621	.896
	Middle phalanx	*Subcutaneous*	.745	.514	.866
		Deep	.843	.676	.921
	Proximal phalanx	*Subcutaneous*	.907	.822	.951
		Deep	.889	.790	.942
	MTP joint	*Subcutaneous*	.795	.610	.893
		Deep	.740	.508	.863
	Metatarsal bone	*Subcutaneous*	.950	.905	.974
		Deep	.912	.793	.958
**Digit V**	Distal phalanx	*Subcutaneous*	.945	.895	.971
		Deep	.767	.553	.878
	Middle phalanx	*Subcutaneous*	.857	.712	.927
		Deep	.719	.313	.870
	Proximal phalanx	*Subcutaneous*	.784	.591	.887
		Deep	.866	.745	.929
	MTP joint	*Subcutaneous*	.900	.808	.947
		Deep	.837	.692	.914
	Metatarsal bone	*Subcutaneous*	.892	.794	.943
		Deep	.942	.889	.969

* The level of agreement was categorized as follows: 0.0 = poor, 0.01-.20 = slight, 0.21-.40 = fair, 0.41-0.60 = moderate, 0.61-0.80 = substantial, 0.81-1.00 = almost perfect agreement.
CL, confidence limit; ICC, intraclass correlation coefficient; MTP, metatarsophalangeal (joint).

Supplementary Table 5: Intra-reader agreement Reader 2.

			ICC *	Lower 95% CL	Upper 95% CL
**Digit I**	Distal phalanx	*Subcutaneous*	.892	.724	.951
		Deep	.917	.842	.956
	Proximal phalanx	*Subcutaneous*	.875	.762	.934
		Deep	.945	.893	.971
	MTP joint	*Subcutaneous*	.915	.839	.956
		Deep	.889	.699	.951
	Metatarsal bone	*Subcutaneous*	.908	.825	.951
		Deep	.842	.696	.918
**Digit II**	Distal phalanx	*Subcutaneous*	.840	.696	.916
		Deep	.810	.596	.905
	Middle phalanx	*Subcutaneous*	.773	.566	.881
		Deep	.740	.502	.864
	Proximal phalanx	*Subcutaneous*	.930	.866	.963
		Deep	.885	.763	.942
	MTP joint	*Subcutaneous*	.838	.670	.918
		Deep	.761	.543	.875
	Metatarsal bone	*Subcutaneous*	.919	.845	.958
		Deep	.768	.562	.878
**Digit III**	Distal phalanx	*Subcutaneous*	.863	.646	.938
		Deep	.658	.357	.819
	Middle phalanx	*Subcutaneous*	.811	.546	.912
		Deep	.702	.434	.843
	Proximal phalanx	*Subcutaneous*	.843	.700	.918
		Deep	.801	.622	.895
	MTP joint	*Subcutaneous*	.787	.593	.888
		Deep	.657	.247	.834
	Metatarsal bone	*Subcutaneous*	.882	.776	.938
		Deep	.769	.557	.879
**Digit IV**	Distal phalanx	*Subcutaneous*	.958	.920	.978
		Deep	.689	.407	.837
	Middle phalanx	*Subcutaneous*	.796	.612	.893
		Deep	.752	.533	.870
	Proximal phalanx	*Subcutaneous*	.910	.829	.953
		Deep	.879	.770	.937
	MTP joint	*Subcutaneous*	.788	.595	.889
		Deep	.785	.593	.887
	Metatarsal bone	*Subcutaneous*	.948	.901	.973
		Deep	.873	.754	.934
**Digit V**	Distal phalanx	*Subcutaneous*	.914	.836	.955
		Deep	.704	.357	.855
	Middle phalanx	*Subcutaneous*	.821	.657	.906
		Deep	.732	.487	.860
	Proximal phalanx	*Subcutaneous*	.718	.468	.852
		Deep	.873	.755	.934
	MTP joint	*Subcutaneous*	.899	.806	.947
		Deep	.789	.597	.889
	Metatarsal bone	*Subcutaneous*	.914	.837	.955
		Deep	.933	.872	.965

* The level of agreement was categorized as follows: 0.0 = poor, 0.01-0.20 = slight, 0.21-0.40 = fair, 0.41-0.60 = moderate, 0.61-0.80 = substantial, 0.81-1.00 = almost perfect agreement.
CL, confidence limit; ICC, intraclass correlation coefficient; MTP, metatarsophalangeal (joint).
